# Meeting report: Flux-independent signaling by ionotropic receptors: Unforeseen roles, complexities, and challenges

**DOI:** 10.1016/j.jbc.2022.102330

**Published:** 2022-07-31

**Authors:** Pavel Montes de Oca Balderas

**Affiliations:** Unidad de Neurobiología Dinámica, Dpt. de Neuroquímica, Ciudad de México, Mexico

**Keywords:** flux-independent, signaling, channels, ionotropic receptors, glutamate receptors, VGCC, nAChR, Kv, NMDAR, CNS, central nervous system, LTD, long-term depression, NMDAR, N-methyl D-aspartate receptor, VGCC, voltage-gated calcium channel

The receptors that mediate neurotransmission were organized by the end of the 1970′s into ionotropic and metabotropic receptors by the Nobel laurate Jhon Eccles and his colleague Patrick McGeer (1). Nevertheless, almost 2 decades later, it was reported that the AMPA glutamate receptor elicits what has been called metabotropic-like, noncanonical, nonconducting, nonionotropic or flux-independent activities (2). In the following years, other glutamate receptors were also found to elicit this kind of signaling in the central nervous system (CNS) context, the kainate receptor first and then the N-methyl D-aspartate receptor (NMDAR) (3, 4, 5). Afterward, other relevant receptors were found to behave in a similar manner; they somehow were not aware of the taxonomy that helped us neuroscientists to understand the nervous system function and the synapse. Nonetheless, flux-independent signaling by ionotropic receptors has helped to understand conundrums of neuroscience such as the mechanisms of long-term depression (LTD) or neuronal excitotoxicity. Moreover, these activities of ionotropic receptors have also been described in cells beyond neurons and the CNS and have been involved in some pathologies.

Last summer, in 2021, the *American Society for Biochemistry and Molecular Biology* (ASBMB) sponsored the first symposium on flux-independent signaling by ionotropic receptors: unforeseen roles, complexities, and challenges. This symposium was held virtually on the 21st of June given the current severe acute respiratory syndrome coronavirus 2 pandemic conditions. The main goal of this event was to gather those independent and scattered efforts carried out by different groups through decades that have demonstrated the mechanism of flux-independent signaling by ionotropic receptors. This in turn should enhance the awareness of its relevance in different scientific fields beyond neuroscience including cellular and molecular biology, biochemistry, and immunology, among others, in which flux-independent channel function may open new perspectives beyond electrophysiology to understand cell function. This is due to the ubiquitously expression of channel receptors in cells that do not elicit action potentials, within and beyond the CNS. At the same time, these fields could provide novel approaches to further investigate this mechanism that has been mostly disregarded but that could be critical to expand our understanding of cell physiology and pathology.

In this report, the most relevant aspects of this symposium are briefly highlighted, referring the most recent published works and including the research by some of the scientists who did the first observations with glutamate ionotropic receptors and have continued their investigations. Also, recent findings by young investigators are included, regarding how flux-independent signaling by glutamate ionotropic receptors mediate neuronal function in health and disease. In addition, it is depicted how voltage-gated calcium channels (VGCCs) are proposed to mediate different excitation coupled mechanisms through flux-independent signaling. Finally, it is stressed how other non-neuronal cells also express different ionotropic channels such as the nicotinic acetylcholine receptor, the voltage-gated potassium channels (Kv), or the NMDAR that through flux-independent signaling regulate cell function.

In the morning session chaired by Dr Karen Zito from University of California at Davis, Dr Juan Lerma (Instituto de Neurociencias CSIC-UMH, Spain), who has authored one of the first comprehensive reviews on noncanonical signaling by ion channels (6), recapitulated his now decades long work showing flux-independent signaling by the kainate type glutamate ionotropic receptor, how it mediates different neuronal functions, as well as his most recent findings implicating how a specific intracellular domain of this receptor is involved in G protein signaling. The second talk of this session was lectured by Dr Daphne Atlas (The Hebrew University of Jerusalem, Israel), who also showed her decades-long work demonstrating how flux-independent signaling by VGCCs mediates excitation-contraction, excitation-secretion, and excitation-transcription mechanisms. She explained how her work has provided a temporal framework, suggesting that channel conformational changes enable the fast relationship between VGCC Ca^2+^ binding and functional outcomes (7). The third talk of this session was given by Dr Michel Salter (Hospital for Sick Children, Canada), who also recapitulated his decades long work demonstrating how glycine elicits flux-independent signaling by the NMDAR that modulates its endocytosis, he explained some of the intracellular mechanisms involved, as well as his most recent observations demonstrating how this mechanism affects only NMDAR with GluN1 subunits lacking exon 5 (8). The last talk of this session was lectured by Dr Kim Dore (University of California at San Diego, USA), a young researcher who has coauthored several works with Dr Roberto Malinow, who did some of the early observations indicating flux-independent signaling by the NMDAR regulating its synaptic traffic (9). Dr Dore spoke about her work describing how flux-independent signaling by the NMDAR involves conformational changes and posttranslational modifications of signal transducers and how the former could be related with PSD-95 effects on LTD and conditions such as Alzheimer disease (10).

The second session of the symposium was chaired by Dr Michel Salter from the Hospital for Sick Children. In the first talk Dr Roger Thompson (University of Calgary, Canada) explained his findings regarding how flux-independent signaling by the NMDAR is involved in excitotoxicity, how it modulates Src kinase activity that in turn modulates Ca^2+^ flux through pannexins that occurs at resting membrane potentials (11). He also showed how this signaling is regulated by amyloid beta. In the second talk, Dr Karen Zito (University of California Davis, USA) explained how flux-independent signaling by the NMDAR elicits intracellular pathways that mediate neuronal spine shrinkage, as well as LTD. She also showed that long-term potentiation depends upon this signaling together with Ca^2+^ permeated by the NMDAR that nevertheless may be replaced by Ca^2+^ permeating through VGCC. She also showed how these findings may be relevant for schizophrenia (12). In the third talk, Dr Jesper Sjostrom (McGill University, Canada) showed his results indicating that presynaptic NMDAR use flux-independent signaling to modulate synaptic activity and detailed some intracellular signal transducers involved. He pointed out that these findings may help to understand early observations reporting timing-dependent LTD (13). In the last talk of this session, Dr Robert Bonin (University of Toronto, Canada) showed his results indicating that flux-independent signaling by the NMDAR is involved in pain-induced plasticity at the spinal cord and how it mediates depotentiation that is related with the reversal of hyperalgesia and described some of the intracellular mechanisms involved.

After the lunch break, we had the presentation of some posters from different labs showing flux-independent or putative flux-independent signaling by different ionotropic channels. The last session of this symposium, chaired by Dr Juan Lerma from Instituto de Neurociencias CSIC-UMH, was dedicated to flux-independent signaling by ionotropic receptors in non-neuronal cells. In the first talk, Dr Veronika Grau (Justus-Liebig-Universität, Germany) explained his findings that described how flux-independent signaling by the nicotinic acetylcholine receptor in monocytes modulates interleukin-1 secretion through alternative agonists such as phosphocholine. She also showed some of the intracellular mediators that are involved in this mechanism (14). The second talk was given by Dr Maria Teresa Perez Garcia (Universidad de Valladolid, Spain), who showed how Kv channels modulate cell proliferation in a voltage-dependent and flux-independent manner, related with receptor conformational changes. She also showed the intracellular signaling associated with this effect that involves Kv-specific intracellular domains, MAP kinases, and scaffolding proteins (15). In the last talk of this session and the symposium, this author explained how flux-independent signaling by the NMDAR in cultured astrocytes mediates Ca^2+^ release from intracellular pools after sensing acidic extracellular pH and how it modulates, in combination with flux-dependent signaling, mitochondria membrane potential through the establishment of plasma membrane–mitochondria bridges that allow mass transfer from plasma membrane to mitochondria, mechanisms that could be critical for astrocyte activation in brain pathology but also probably for cell physiology at discrete domains (16).

**Figure 1:**
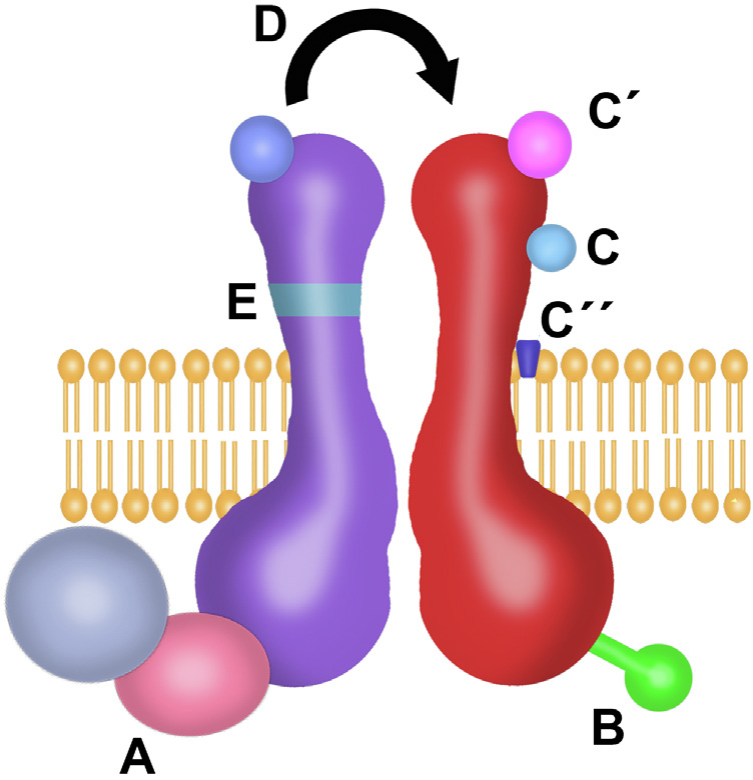
**Molecular mechanisms involved in flux-independent signaling by ionotropic receptors.***A*, the interactome associated with ionotropic receptors is activated through conformational changes that do not open the channel pore but initiate intracellular transduction pathways with molecular partners directly associated but also perhaps with those indirectly associated as part of supramolecular assemblies. *B*, posttranslational modifications (PTM) of ionotropic receptor subunits, such as tyrosine phosphorylation or dephosphorylation, is involved in eliciting flux-independent intracellular signal transduction. *C*, ions, coagonists (C′), or other molecules (C″) alone are capable to elicit flux-independent intracellular signal transduction and unique intracellular outcomes. *D*, information transfer among ionotropic receptor subunits has been involved by different works in flux-independent signaling (Reviewed for NMDAR in (20)). *E*, the lack of certain amino acid sequences in ionotropic receptor subunits may elicit specific intracellular flux-independent signaling and cellular outcomes in response to coagonists alone.

Finally, to close the symposium, conclusions and final comments were made to underpin the most relevant aspects related to flux-independent signaling by ionotropic receptors. Perhaps the most relevant assumption that stems form these findings is the paradigm shift in which ionotropic receptor–mediated signaling is not a linear phenomenon depending necessarily upon ion-flux; instead, it is suggested that ion-flux is one of different possible outcomes from receptor conformational rearrangement. In addition, some molecular mechanisms relevant for this signaling were summarized and include the receptor interactome, posttranslational modifications, or modulators, among others (Fig. 1). One of the key relevant pending questions that was mentioned by different speakers throughout the symposium is that of the evolution of this kind of signaling in ionotropic receptors. It is currently not known if whether flux-independent signaling preceded ionotropic channel function in these molecules, or inversely, or whether they may have simultaneously emerged along with these receptors. In this regard, the observations made with GluND receptors (17, 18) and the evolution of glutamate ionotropic receptors (19) may provide clues to answer this question. Finally, it was suggested that the term “flux-independent” could be used to systematically refer this kind of signaling, since a plethora of different terms have been employed to describe it through decades. This homogenization could help the field to advance more consistently, by easing literature reference, indexing, search, and providing some cohesion to the somewhat disjoined efforts of different labs in different continents in the last decades that have challenged neuroscience paradigms.

Hopefully, this symposium will also cooperate to setup a new framework to understand ionotropic receptors, their biology, biochemistry, biophysics, and signaling, etc., that in turn will help to boost our comprehension of the cell in health and disease, in the CNS but also beyond the CNS, where channels are ubiquitously expressed but are poorly considered, despite they are there.
